# A comparison of the multiple oocyte maturation gene expression patterns between the newborn and adult mouse ovary

**Published:** 2013-10

**Authors:** Soghra Bahmanpour, Tahereh Talaei Khozani, Nehleh Zarei fard, Mansoureh Jaberipour, Ahmah Hosseini, Tahereh Esmaeilpour

**Affiliations:** 1 *Laboratory for Stem Cell Research, Department of Anatomy, School of Medicine, Shiraz University of Medical Sciences, Shiraz, Iran.*; 2 *Institute Cancer Research, School of Medicine, Shiraz University of Medical Sciences, Shiraz, Iran.*

**Keywords:** *Ovarianfollicle*, *Ovary*, *Geneexpression*, *Mice*, *Meiosis*

## Abstract

**Background:** The interaction between follicular cells and oocyte leads to a change in gene expression involved in oocyte maturation processes.

**Objective: **The purpose of this study was to quantify the expression of more common genes involved in follicular growth and oocyte developmental competence.

**Materials and Methods:** In this experimental study, the expression of genes was evaluated with qRT-PCR assay in female BALB/c mice pups at 3-day of pre-pubertal and 8 week old virgin adult ovaries. The tissue was prepared by H&E staining for normal morphological appearance. The data were calculated with the 2-∆Ct formula and assessed using non-parametric two-tailed Mann-Whitney test. The p<0.05 was considered as significant.

**Results: **The data showed a significant increase in the level of Stra8 and GDF9 in adult compared with newborn mice ovaries (p=0.049). In contrast, a significant decrease in the level of Mvh, REC8, SCP1, SCP3, and ZP2 was observed in adult mice ovaries compared to those in the newborn mice ovaries (all p=0.049 except SCP1: p=0.046). There was no significant difference in the level of OCT4 and Cx37 expression between adult and newborn mice ovaries.

**Conclusion:** The modifications in gene expression patterns coordinate the follicular developmental processes. Furthermore, the findings showed higher expression level of premeiotic gene (Stra8) and lower level of meiotic entry markers (SCP1, SCP3, and REC8) in juvenile than newborn mouse ovaries.

This article extracted from Ph.D. thesis. (Nehleh Zarei fard)

## Introduction

Growth and development of primordial follicles to ovulatory status depends on cellular interactions with granolusa cells along with changes in oocyte gene expression. This interaction is regulated by gap junction molecules such as connexin 37 (Cx37), which is a precondition for foliculogenesis (-). Connexin-37-deficient mice cannot ovulate and fail to have nuclear and cytoplasmic meiotic competence (4). In mice, oocytes complete prophase during the ﬁrst week after birth and a pool of primordial follicles formed which are surrounded by a single layer of follicular cells arrested in dictyate stage of the first meiotic prophase (5-7). 

Beyond the dictyate stage of female germ-cell development, the formation of synaptonemal complex proteins (SCP1 and SCP3) is not detectable (5, 8). Meiotic recombination protein REC8 is also decreased after birth and disappears in mice chromosomes gradually as they age (9-11).

OCT4 (octamer-binding transcription factor) expression that is down-regulated with the onset of meiosis, once up-regulated when the oocytes were formed within the primordial follicles (12, 13). Moreover, the strongest expression of Mvh (Mouse Vasa homologue) was detected in the cytoplasm of primordial oocytes (14, 15). Growth differentiation factor-9 (GDF-9) is secreted by oocytes throughout follicular growth. GDF9 expression is involved in follicle formation through modulation of granulosa cell development and function. Lack of GDF9 expression in mice causes follicular development arrested at the primary stage, leading to infertility (16). 

Granulosa cells provide a critical microenvironment for follicular growth by producing several growth factors along with changes in zona pellucida (ZP) protein gene expression (2). Zona pellucida has a critical role in reproduction phases including folliculogenesis, fertilization and implantation (-). Stra8 (stimulated by retinoic acid gene 8) is expressed in premeiotic germ cells and induces their meiotic entry (20). 

Analysis of Stra8 in ovaries of young female mice indicated that expression of this gene was increased in the ovaries of young female mice paired with either young male or aged male mice and expression level of the gene was age-dependent (        21 , 22). It seems that these findings provided a new oocyte resource in postnatal mammalian ovary (23, 24). In addition, there are many investigations focusing on gamete differentiation from stem cells (-). 

To standardize in vitro germ cell differentiation, it is needed to compare the gene expression pattern with in vivo gamete development. The similarity of gene expression pattern during germ cell development in vivo and in vitro is a critical criterion that should be evaluated for developmental competence of stem cells differentiation toward oocytes with characteristic of primordial or antral follicle. Review of the literature showed a limited number of researches focusing on comparing the gene expression pattern of the oocytes at various developmental stages. 

It is necessary to understand the gene expression patterns during in vivo ovarian development for fertilization competence assessment. The aim of this study was to compare the expression of the most common and important genes involved in oocyte development (OCT4, Mvh), progression into meiosis (SCP1, SCP3, REC8) and maturation (GDF9, ZP2, Cx37) in neonatal and adult mice.

## Materials and methods

**Animals**

This study was an experimental intervention. The animal handling was according to the rules of the Ethics Committees of Shiraz University of Medical Sciences. Female BALB/c mice pups at 3-day of pre-pubertal (n=15) and 8 weeks old virgin adult mice (n=5) were purchased from the Animal House of Shiraz University of Medical Sciences. The mice were kept at a temperature- and light-controlled environment (12L: 12D), and were provided with food and water ad libitum. 

The ovaries of the newborn and adult mice were removed and collected after euthanizing via chloroform and cervical dislocation, respectively. For morphological evaluation, the ovaries were stained with hematoxylin and eosin (H&E). Real-Time Quantitative Reverse Transcription PCR (qRT-PCR) was done to investigate the expression of genes between adult and newborn mouse ovaries.

**Tissue sample staining**

For morphological evaluation, adult and newborn ovaries were fixed in 10% formalin and Bouin's solution, respectively. After dehydration and paraffin embedding, the tissues were serial sectioned with 5 µm thickness and stained with H&E. 

**RNA isolation and reverse transcription**

Total RNA of the ovaries was extracted by Trizol reagent (Invitrogen, Germany) according to the manufacturer’s instructions. Briefly, after cell lysing by Trizol reagent, chloroform was added to each tube. Samples were centrifuged and RNA in the upper aqueous phase was pelleted by 100% isopropanol. The RNA pellet was washed by 75% ethanol and eluted in the DEPC-treated water. 

The quantity and quality of the extracted RNA samples were estimated by spectrometry at 260 and 280 nm. To avoid DNA contamination, RNA was treated with DNase I (Invitrogen-Gibco, Paisley, UK) before cDNA synthesis. cDNA was synthesized from 5 µg of extracted RNA by RevertAid First Strand cDNA Synthesis Kit (Fermentas, Vilnius, Lithuania) according to manufacturer’s instructions. 

**Real-Time Quantitative Reverse Transcription PCR** **(qRT-PCR)**

The quantities and the expression of ovarian genes were determined with a Bio-Rad system (Chromo4 Real-time PCR Detector, Bio-Rad, Foster City, CA, USA) for qRT-PCR and SYBER Green PCR Master Mix (Applied Biosystems, Foster City, CA, USA). Each PCR reaction was carried out in a final volume of 20 μL containing 2µL cDNA (0.5 μg), 0.3 µL of each primers (150 nmol, Table I), 10 µL of SYBR Green I PCR Master Mix, 7.4 µL RNase-free water. RT-PCR amplification was done in 40 cycles using the following program: denaturation at 95^o^C for 15 s, annealing at 57^o^C for 30s and extension at 60^o^C for 60 s. The QRT-PCR amplification products were examined by melting curve analysis. Expression of the ß-actin was used as a housekeeping gene to normalize the level of target gene expression. 

**Statistical analysis**

All data are expressed as mean±SD of at least three independent experiments. The data were analyzed using Mann-Whitney test. The relative amounts of all genes were determined from 2^-∆Ct^. Relative expressions were plotted and evaluated using Graph Pad Prism 5. P<0.05 was regarded as significant.

## Results

**Figure 1:**
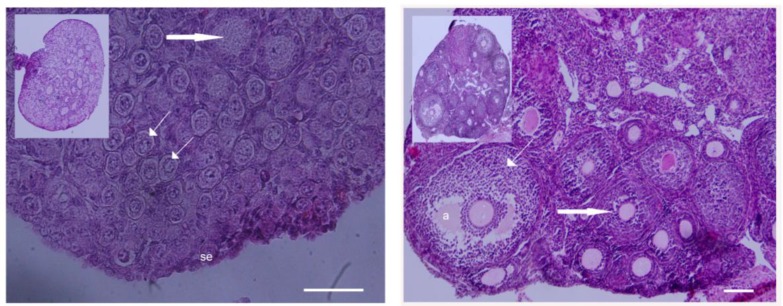
Microphotographs of 3 dpp and adult mouse ovaries. Sections were stained with H&E for identification of follicular status. A; Newborn mouse ovary with primordial and primary follicles. Thick and thin arrows are primary and primordial follicles, respectively. B; Adult mouse ovary with preantral and antral follicles. Thick and thin arrows are preantral and antral follicles respectively. Abbreviation: a, antrum; se, surface epithelium. Scale bar= 50µm

**Figure 2:**
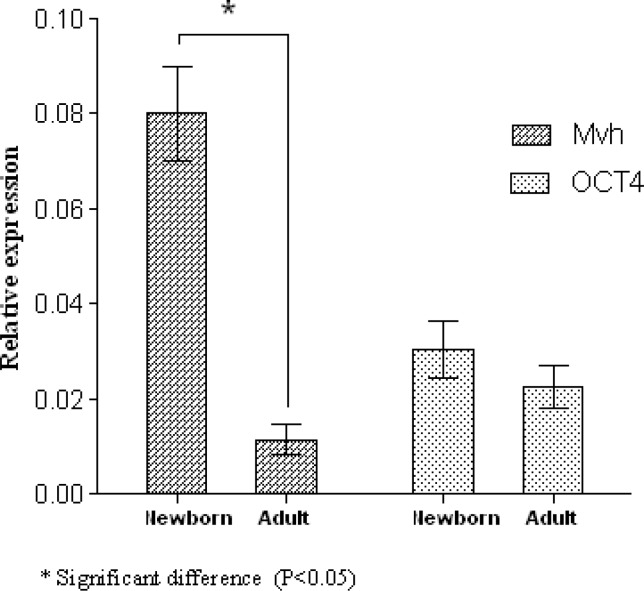
OCT4 and Mvh gene transcripts in the newborn and adult mice. The data presented as mean±SD (n= 3).

An analysis of the gene expression in ovary has been studied at two different stages of development. Hematoxylin and eosin-stained sections from the ovaries showed the presence of primordial and primary follicles at 3 days post-partum (dpp) and primordial, primary and antral follicles at 8 weeks of age (Figure 1). The qRT-PCR for genes involved at various stages of oocyte maturation including OCT4, Mvh, SCP1, SCP3, Stra8, REC8, ZP2, GDF9, Cx37 was performed in newborn and adult mouse ovaries. A non-significant elevation in OCT4 expression was detected in newborn mice compared with those in adult mice (p=0.089). The expression of Mvh gene was significantly higher in newborn mice than those in adult mice (p=0.049) (Figure 2). Mvh expression increased 6.9-fold in newborn mice compared to adult ones. 

**Figure 3:**
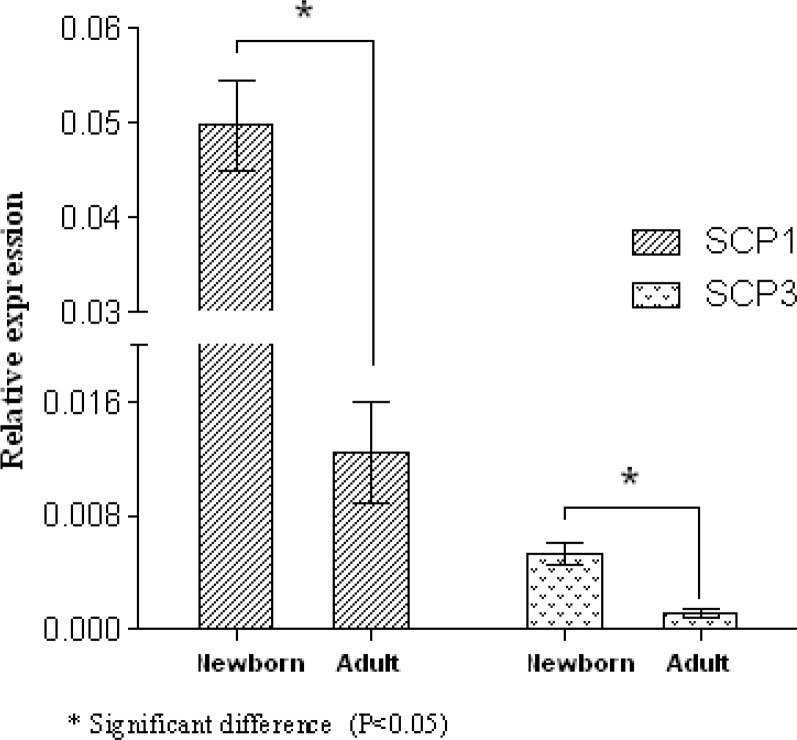
Gene expression levels of SCP1 and SCP3 in newborn and adult mice. The data presented as mean±SD (n= 3).

**Figure 4:**
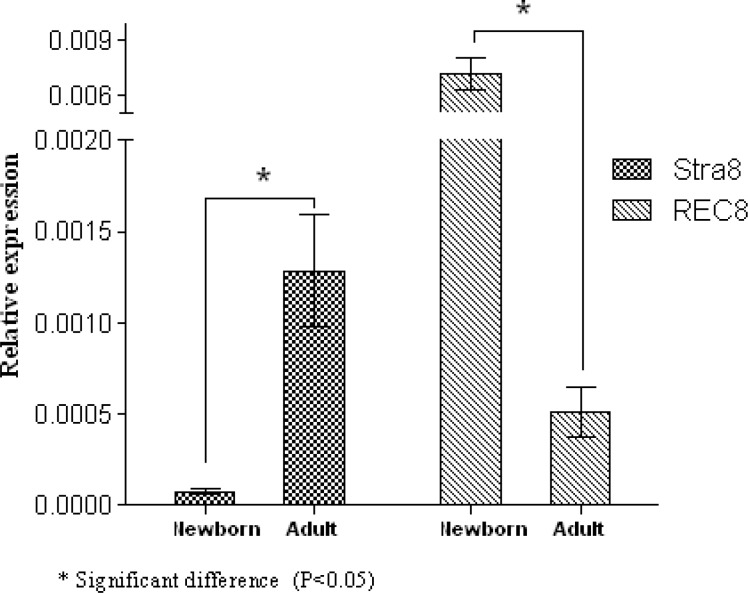
QRT-PCR analysis of normalized Stra8 and Rec gene expression in the newborn and adult mice. The data presented as mean±SD (n= 3).

**Figure 5:**
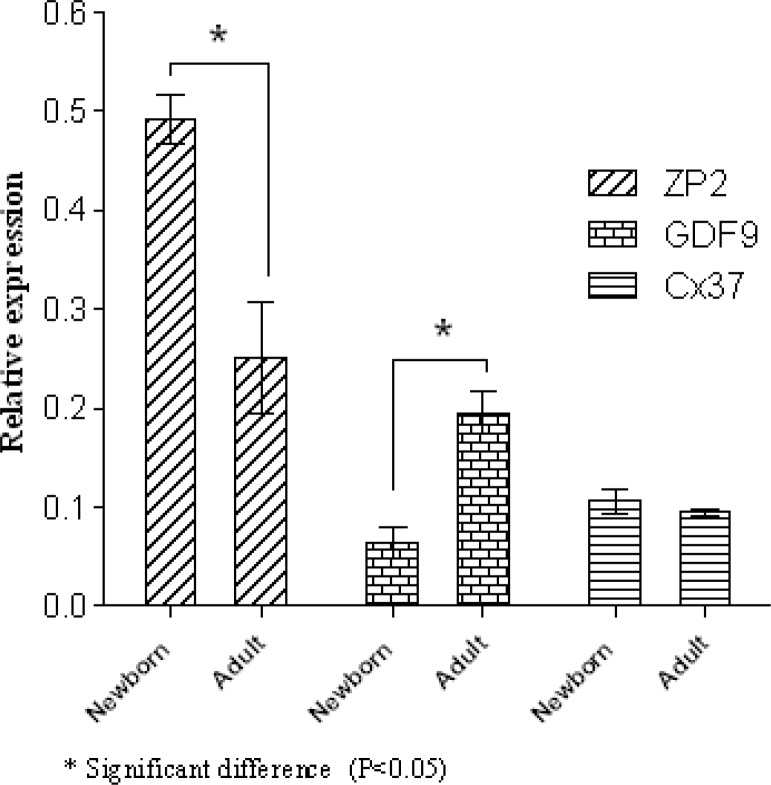
Expression levels of ZP2, GDF9 and Cx37 in newborn and adult mice Data are represented mean±SD (n= 3).

A 4-fold increase in SCP1 and a 4.58-fold increase in SCP3 were detected in the newborn mice when it was compared with adult mice (p=0.046 and p=0.049, respectively) (Figure 3). On the other hand, a 14.45-fold elevation in Stra8 expression was also shown in adult mice compared to newborn ones (p=0.049). In contrast, a significant increase in the expression of REC8 expression (17.48-fold) was detected in newborn mice compared to adult ones (p=0.049) (Figure 4). Newborn mice showed a significant increase (1.68-fold) in ZP2 expression compared with adult mice (p=0.049). In contrast, expression of GDF9 gene was increased 3- fold in adult mice compared with the newborn ones (p=0.049). However, there were no significant differences in Cx37 gene transcript between newborn and adult mice (p=0.82) (Figure 5).

**Table I:** List of the specific forward and reverse primers used for amplification in qRT-PCR

**Gene**	**Forward primer (5’-3’)**	**Reverse primer (5’-3’)**	**Annealing temperature(** ^o^ **c)**
OCT4	GCCAGACCACCATCTGTCGCT	AGGGTCTCCGATTTGCATATCTCCT	57
Mvh	CAAGCGAGGTGGCTGCCAAG	CTGAATCACTTGCTGCTGGTTTCC	57
SCP1	GACAACGGCCCAGGAGGCA	TCTGCGGTTTCACGGCGGA	57
SCP3	GCAGAGAGCTTGGTCGGGGC	CTTTAGATGTTTGCTCAGCGGCTCC	57
Stra8	GCATGAAGGACAGCGGCGTG	AAAGGATCTCTTCTGGGGTGGACTC	57
REC8	GCCCTAGAAGGTGCTGGTTTGG	GTGGGGTCACCTCAGTGAGTAGG	57
ZP2	GCAGTGCCTTGATCTGTAACCAAG	TGGGGTCAACACCTTTGGATGAAG	57
GDF9	CGTCCGGCTCTTCAGTCCCT	CCATCGGCAGCGGTCCTGTC	57
Cx37	GGGCAAGCAGGCGAGAGAG	GCCAGCCCCAGGATGAGGATG	57
Βactin	CCCGCGAGCACAGCTTCTTTG	CCATCACACCCTGGTGCCTAGG	57

## Discussion

Comparison of the gene expression patterns in mice ovaries showed a significant increase in Stra8 and GDF9 levels and a significant decrease in Mvh, Rec8, SCP1, SCP3 and ZP2 levels in adults compared with newborn mice. A non-significant decrease of OCT4 was observed in adult ovary compared to newborn ovary. However, the Cx37 expression level was constant during puberty. Literature showed that upon meiosis arrest, Stra8 disappears and it is not detectable anymore in the newborn ovary except for those cases with oocyte defect at molecular level (20). 

We observed that Stra8 mRNA was detectable at a very low level in newborn ovaries and it increased significantly in young adult mice. A previous data from ordinary RT-PCR showed the lack of Stra8 expression in 8 week old mice caged with the same age female mice (21). On the other hand, the age of the female mice has been reported to impact the Stra8 gene expression and ovarian follicle reservation (22). In vitro development of oocyte-like cells derived from ovarian surface epithelial stem cells has been also reported in post-menopausal women (29). 

Our qRT-PCR showed an increase in Stra8 expression and it may be related to the neo-oogenesis induction in young female mice; it is a rare phenomenon in the ovary (        21 , 30, 31). On the other hand, the difference in genetic strains and species has a marked impact on gene expression of ovaries such as ZP synthesis; therefore, the pattern of Stra8 expression also may be affected by the difference in various strains (17). Morphometry-based analyses revealed a negative relationship between the number of non-atretic primordial and total immature (primordial, primary and preantral) follicles with Stra8 expression. 

The hormonal changes during puberty causes a decrease in the total number of ovarian follicles and it may be responsible for high stra8 gene expression level after adulthood. It has been shown that the level of gene expression of Stra8 is differing from ovary to ovary in adult mice (22, 23). Meiotic recombination protein REC8 is located between sister chromatids along with axial element during meiotic prophase I (11). There is a controversy about the REC8 expression pattern after birth in the literature (10). 

From diplotene onward, REC8 dissociates from axial elements and finally limits around the centromeres from metaphase I. It has been suggested that the amount of REC8 reduces after birth (8). The reduction continued with age gradually and in this way, the incidence of aneuploid eggs increase (10, 11). Immunocytochemistry showed lack of REC8 in the mouse dictyate arrested-oocytes after 8 dpp (9). In the present study, REC8 significantly decreased in adult ovaries compared with newborn mice; however, did not disappear completely.

Our data showed that SCP1 and SCP3 expression, a component of central element of synaptonemal complex and lateral element protein of synaptonemal complexes, respectively, exhibited lower expression in adult ovaries as compared with those in newborn mice group (5). Most of the oocytes finish prophase I around the time of birth and SCP1 is normally absent at the end of the first meiotic prophase (5, 32). The absence of elements of synaptonemal complex in the mouse oocyte has been detected at the ﬁrst week of postnatal life; however, our data indicated existence of this protein at 3 dpp (33).

 A high decline in the expression of SCP1 gene 48 hours after the birth in rat ovaries has been also reported (7). SCP3 is required for mouse chromosomal compaction at zygotene and pachytene stages but not for sister-chromatid cohesion, homologue alignment or synapsis (34). SCP3 is not present at the centromere during the ﬁrst meiotic division in females (33). However, the persistence of lateral element protein SCP3 at MI, and even MII, has been reported in human (35). Germ line stem cells from the surface epithelium of juvenile and adult ovaries have been found to express SCP3 and Mvh; however, these cells do not have the ability to further develop (36). This finding may account for the low level of SCP1 and SCP3 expression in adult ovaries in our experiment.

According to the study of isolated oocytes on the basis of oocyte diameter, the expression of ZP2 (zona pellucida 2) has not been found in resting oocytes and also ovulated eggs during oogenesis. The highest amount of ZP2 was synthesized in 50-60 µm diameter mouse oocytes and it was started to diminish after oocyte diameter reached 65-µm (18). In contrast, detection of ZP2 transcripts in oocytes has been reported before birth and also in resting adult oocytes (15 µm diameter) (19, 37). 

It should also be noted that although previous studies have emphasized that ZP2 was expressed only in the mouse oocytes, the others have detected ZP2 in both oocyte and granulosa cells at different folliculogenesis stages in Kunming adult mice. The amount of ZP2 mRNA transcripts in oocytes declined linearly from primary to mature follicles (17, 18). In the present study, more ZP2 mRNA was generated in whole ovaries in newborns compared to adults. 

Newborn ovary contains small size oocyte in comparison to the adult mice ovary with large size oocyte. The number of granolusa cells in adult ovary increased as compared to those in newborns; and, therefore, the total RNA extraction from adult ovary contains more RNA from granulosa cells compared to RNA from the oocyte. It may lead to a decrease in the total amount of ZP2 mRNA in the extraction. It can also be concluded that ZP2 is produced by oocyte but not by granulosa cell in BALB/c mice. Immunohistochemistry revealed that OCT4 was expressed from oocytes in primordial follicles until fully-grown    (12) . 

Although our results showed a non-significant decrease in OCT4 expression in adult ovary, the qRT-PCR data confirmed the immunohistochemistry findings from previous work that revealed the existence of OCT4 in both newborn and adult mice ovaries (8). A decrease in the number of primordial germ cell and an increase in atretic and antral follicles have been reported at 42 dpp in C57BL/6 adult mouse ovaries      (36) . It can be attributed to the lower expression of OCT4. However, Varras *et al* showed that Oct-4 transcripts, in human, could be detected in luteinized granulosa cells at a ratio of 48% but germ cell markers (DAZL) were absent    (13) . 

Mvh expression in the hamster oocytes increased in ovaries containing primordial follicles compared to ovaries containing oocyte clusters with undifferentiated somatic cells and oocytes (15-day fetuses) and its expression reflected oocyte maturation in parallel with follicular development (15). Immunocytochemistry of the adult mice ovary also showed the strong intensity of the reaction for Mvh in the cytoplasm of primordial oocytes and its expression decreased with the onset of follicular maturation so that Mvh disappeared completely in the oocyte of antral follicles (14). 

The data from the present study showed that the enhancement of Mvh expression in newborn ovary compared with adult ovary may be attributed to the oocyte maturation process. In most of the investigations, the gene expression was studied without checking the time of collection of ovary from females and it can influence the results. The controversy that has been observed in the literature may be attributed to the difference in the age of the mice and to the genetic background of various mouse strains (        23 , 36). The isolation of RNA of the newborn and adult mouse ovaries can be used to improve our knowledge about expression of genes in two developmental stages and also provides a standard genetic tool to understand in vitro developmental competence of stem cell differentiation toward the oocyte (-).
